# Impact of COVID-19 on quality of life in survivors with pulmonary sequelae

**DOI:** 10.1038/s41598-024-57603-z

**Published:** 2024-03-22

**Authors:** Irene Rodríguez-Galán, Natalia Albaladejo-Blázquez, Nicolás Ruiz-Robledillo, José Francisco Pascual-Lledó, Rosario Ferrer-Cascales, Juan Gil-Carbonell

**Affiliations:** 1 Pneumology Department, Alicante General University Hospital—Alicante Institute of Health and Biomedical Research (ISABIAL), 03010 Alicante, Spain; 2 Department of Health Psychology, University of Alicante, 03690 Alicante, Spain

**Keywords:** SARS-CoV-2 pneumonia, COVID-19, Health-related quality of life, Sequelae, Life quality, Pulmonary fibrosis, Health care, Medical research

## Abstract

SARS-CoV-2 respiratory infection is still under study today, mainly because of its long-term effects. This study aims to analyse health status and health-related quality of life (HRQoL) in survivors of coronavirus pneumonia (COVID-19) who have developed pulmonary sequelae. Prospective observational study of patients diagnosed with COVID-19 pneumonia between February and May 2020. Reviews were conducted at 3 and 12 months after hospital discharge. HRQoL was assessed by administration of the SF-36 questionnaire and data related to medical records and physical examination were also collected. In addition, chest X-ray, computed tomography and pulmonary function test were included as additional tests. 305 patients were admitted for COVID-19 pneumonia of which 130 (42.6%) completed follow-up. The mean age of the enrolled group was 55.9 ± 15.9 years. The most prevalent persistent symptoms were dyspnea (37.3%) and asthenia (36.9%). Pulmonary sequelae were detected in 20.8% of participants. The most frequent alteration was ground ground glass opacities (GGO) (88.9%), with mild extension. Fibrotic changes were found in only 2% of cases. When comparing the two groups, at 3 and 12 months of evolution, lower scores in the vitality (VT) and mental health (MH) domains were found only in the group without sequelae. Days of hospitalisation and Charlson index acted as influential factors on HRQoL. Minimal or mild pulmonary sequelae of SARS-CoV-2 do not cause further deterioration of HRQoL. Repeated medical care and pulmonary rehabilitation are effective tools to improve HRQoL.

## Introduction

The COVID-19 pandemic, with more than 774,000,000 confirmed cases^1^, is a health problem that has been present in society for the past 4 years. During this period, research has been conducted on its sequelae, with concerns about the impact on patient’s health-related quality of life (HRQoL) and lung function^2–5^.

Large series of patients, with more than 70,000 cases analysed, describe that the usual presentation of the disease is asymptomatic or in the form of mild pneumonia (81%)^6^. Severe pneumonia with respiratory failure and adult respiratory distress syndrome (ARDS) accounts for 5% of cases^6^, and although clinical and radiological improvement usually occurs during the course of the disease, high-resolution computed tomography (HRCT) studies performed in the acute phase already show signs of interstitial involvement^7^. Several predictors for the development of fibrotic lung disease have been identified, including older age, male gender, duration and severity of acute disease, radiological extent, need for mechanical ventilation and prolonged hospitalisation^8–13^.

However, the evolution of lung lesions caused by SARS-CoV-2 remains poorly defined in the literature. Recently, several studies on radiological changes during the healing process of COVID-19 pneumonia have been published and describe persistence of abnormalities such as pleuroparenchymal bands, linear atelectasis, bronchiectasis and/or bronchiolectasis in 2 to 24% of cases 1 year after the initial diagnosis^14,15^.

Previously, with the severe acute respiratory syndrome (SARS) epidemic of 2003, varying degrees of pulmonary fibrosis^16,17^ and impaired lung function up to 5 years after the acute episode were described^18^. These cases were associated with impaired HRQoL, with decreased scores in most domains^19^.

Those who have survived SARS-CoV-2 pneumonia have been shown to have a poorer quality of life than the general population^2,20,21^. At disease onset (< 4 weeks post-infection), greater deterioration in the physical component is observed, especially in severe cases, women, elderly and low-income patients^3^. This deterioration persists in the stable phase (> 4 weeks post infection) in up to 59% of patients^4^ and, although there is apparent improvement over time, lower scores are still reported in all HRQoL domains after 1 year, except for mental health^2^.

Regarding the treatment, corticosteroid therapy has changed the therapeutic paradigm in the acute phase, after demonstrating that its use for 3 weeks in patients with organizing pneumonia produces clinical (dyspnea), functional (increase in FVC% pred and DLCO% pred), physiological (increase in distance travelled in 6 minutes), and radiological (ground glass opacities and consolidation) improvements. However, there is insufficient evidence to recommend its use in chronic post-COVID syndrome^5^. Clinical trials are ongoing to assess the optimal duration of corticosteroids and the safety and efficacy of antifibrotic drugs. Currently, the main clinical practice guidelines recommend multidisciplinary rehabilitation (physical, psychological and psychiatric) as a pillar of management and treatment of patients with sequelae, including lung damage^22,23^.

Therefore, the purpose of this study was to assess the impact of pulmonary sequelae on HRQoL and its evolution over time (at 3 and 12 months post-infection) in patients who have survived bilateral SARS-CoV-2 pneumonia. Studies are needed to increase knowledge about the sequelae after COVID-19 infection and to improve patient care and management.

## Materials and methods

### Study design and participants

This prospective observational study follows patients who survived COVID-19 pneumonia between February and May 2020 at the Hospital General Universitario Dr. Balmis, Alicante. Inclusion criteria were confirmed SARS-CoV-2 infection and pneumonia evidenced by radiological study. For confirmation of infection, the sample was collected by nasopharyngeal aspirate or lower respiratory tract sampling and the technique used was real-time reverse transcription-polymerase chain reaction (RT-PCR). The diagnosis of pneumonia was made by analysis of the patient's symptoms and a chest X-ray. All subjects are over the age of majority (18 years old).

### Procedure

The first visit took place 12 weeks after discharge from hospital for COVID-19 pneumonia. Demographic, radiological and spirometric data were collected. The participants with FVC < 80%, FEV1/FVC < 70%, respiratory failure and/or residual radiological lesions on the chest X-ray, were referred to the specialized pulmonology clinic.These participants underwent a chest CT scan 24 weeks after the initial episode and more comprehensive functional tests such as lung diffusion. In addition, in these patients all tests were repeated at 12 months including spirometry, lung diffusion and chest CT. At the first visit, the SF-36 questionnaire was also self-administered and after 12 months it was delivered electronically (via email or phone call). All patients with pulmonary sequelae were referred to the rehabilitation service. The rehabilitation programme used in our centre included muscle training, education and respiratory physiotherapy. The duration was between 8 and 20 sessions with a frequency of 2 to 5 sessions per week. In detail, for muscle training, aerobic or resistance training was carried out using a cycloergometer or treadmill for approximately 20 minutes, strength training with weightlifting exercises and respiratory muscle training with an Inspir type respiratory stimulator. Education was mainly based on self-care. Basic anatomy and physiology of the lung and breathing, symptom management and healthy lifestyle habits were taught. Respiratory physiotherapy in these patients was aimed at respiratory re-education techniques to re-educate the ventilatory pattern, prevent thoracic deformation, promote energy saving and reduce the sensation of dyspnoea and relaxation techniques.

**Figure 1:**
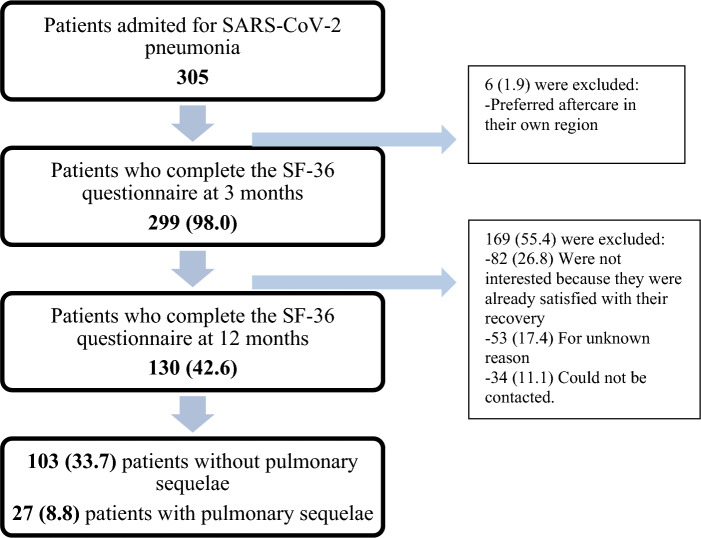
Flow chart of the study. The data are expressed in absolute numbers and percentages for the referring to the whole group.

In total, 305 patients were treated and discharged with a diagnosis of COVID-19 pneumonia between February and May 2020. Of these, 130 (42.6%) completed follow-up. Details of why follow-up was discontinued are shown in Fig. 1.

The study was carried out taking into account the recommendations formulated by the Spanish society of pulmonology and thoracic surgery (SEPAR) for the care and monitoring of post-COVID-19 patients^24^. The study has approval from the Research Medication Ethics Committee of the Alicante Health Department. All participants signed their consent to participate in the study.

### Measures

#### Sociodemographic and clinical data

Demographic and clinical data were obtained by reviewing the medical history. Data included comorbidities (smoking, high blood pressure, hypercholesterolemia, diabetes, obesity, immunosuppression, COPD, asthma and/or other respiratory diseases), and clinical signs and symptoms. A physical examination with oximetry and pulmonary auscultation was performed and as complementary tests a chest X-ray, spirometry and the SF-36 health questionnaire were completed. Participants with radiological abnormalities also underwent a chest CT scan. The severity of the radiological involvement during admission and in the review was evaluated with an adapted scale that assessed the involvement of the extension in the anteroposterior radiograph from 0 to 10^25^. The radiological findings visualized in the thoracic CT were defined following the glossary of terms for thoracic imaging^26^. The extent of COVID-19 pneumonia abnormalities was quantified as follows: for each of the five lobes, lung involvement was reported as none (0%, score 0), minimal (1–25%, score 1), mild (26–50%, score 2), moderate (51–75%, score 3), or severe (76–100%, score 4)^14^. Radiological sequelae were considered to be the appearance of ground glass opacities (GGO), fine and/or coarse linear opacities, reticulation, traction bronchiectasis/traction bronchioloectasis, honeycombing and mosaic, visualised on chest CT at 24 weeks (6 months) after COVID-19 pneumonia. Spirometry was interpreted according to the reference values included in the documents of the American Thoracic Society (ATS) and the European Respiratory Society (ERS)^27^.

### SF-36 questionnaire

To evaluate HRQoL, the SF-36 health questionnaire, designed by Ware et al., was selected. and adapted to Spanish by Alonso et al.^28^. The questionnaire allows obtaining a generic estimate of HRQOL through 36 scoring items and covers 8 aspects: physical function (PF), physical role (PR), body pain (BP), general health. (GH), vitality (VT), social function (SF), emotional role (RE) and mental health (MH). The higher the score, the better the health status and the score range is from 0 (worst score) to 100 (best score). Additionally, a health transition item and two summary scores can be calculated: the physical summary component (PCS) and the mental summary component (MCS). Completion time is between five and ten minutes. The Cronbach’s alpha internal consistency coefficient of the questionnaire exceeds the minimum value recommended for group comparisons (Cronbach’s α = 0.7) for all scales except the SF^29^. The RP, PF and RE scales present better reliability results, most of the time exceeding the value of 0.90, the recommended limit for individual comparisons^29^.

### Data analysis

Categorical variables were expressed as number (%) and compared using the chi 2 test or Fisher’s exact test. Continuous variables were expressed as mean, standard deviation and range. Student’s *t* test was used to test the significance of comparisons. Also to compare patients with and without pulmonary sequelae after COVID-19 pneumonia and the different domains of the SF-36 questionnaire. To analyse the differences between the functional tests and the domains of the SF-36 questionnaire at 3 and 12 months post-infection, a paired samples *t* test was performed. A general linear repeated measures model was used to investigate interactions between factors and the effects of individual factors. In the final group of the study all participants have completed the questionnaire. In the event that some items were missing, the recommendations stipulated in the questionnaire manual were followed in order to deal with the missing data. The authors used SPSS for windows, version 24.0.0.0 (IBM Corp. Published 2016. IBM SPSS Statistics for Windows, version 24.0. IBM Corp., Armonk, NY, USA). A two-sided α of less than 0.05 was considered statistically significant.

## Results

### General characteristics

The study evaluated a total of 130 patients surviving COVID-19 pneumonia. The group with post COVID-19 pulmonary sequelae consisted of 17 males (63%) and 10 females (37%); with a mean (standard deviation) age of 65.3 (9.9) years. Secondly, the group without sequelae consisted of 46 males (44.7%) and 57 females (55.3%); with a mean (standard deviation) age of 53.4 (16.2) years. Table 1 shows the demographic characteristics, comorbidities, and symptoms at 3 months. Age, Charlson index, chest X-ray score and spirometric values are shown in Table 2.

**Table 1:** Qualitative characteristics of the included patients.

Characteristics	Total (N = 130)	Without post COVID-19 sequelae (N = 103)	Post COVID-19 sequelae (N = 27)	*p*
Gender				
Male	63 (48.5)	46 (44.7%)	17 (63.0%)	0.090
Female	67 (51.5)	57 (55.3%)	10 (37.0%)	
Hypertension	50 (38.5)	36 (35.0%)	14 (51.9%)	0.108
Diabetes mellitus	13 (10.0)	11 (10.7%)	2 (7.4%)	1.000
Pulmonary disease (chronic obstructive pulmonary disease, asthma, other)	21 (16.2)	16 (15.5%)	5 (18.4%)	0.770
Immunosuppression	7 (5.4)	5 (4.9%)	2 (7.4%)	0.635
Obesity	38 (29.2)	28 (27.2%)	10 (37.0%)	0.347
Smoking history	60 (46.2)	46 (44.7%)	14 (51.9%)	0.475
Cough	29 (22.3)	25 (24.3%)	4 (14.8%)	0.299
Dyspnea	49 (37.7)	37 (35.9%)	12 (44.4%)	0.394
Asthenia	48 (36.9)	40 (38.8%)	8 (29.6%)	0.388
Myalgias-arthralgias	25 (19.2)	20 (19.4%)	5 (18.5%)	0.930
Anosmia-dysgeusia	31 (23.8)	25 (24.3%)	6 (22.2%)	0.839
Memory loss	14 (10.8)	10 (9.7%)	4 (14.8%)	0.485
Dermatological disorders	12 (9.2)	9 (8.7%)	3 (11.1%)	0.711
Headache	21 (16.2)	17 (16.5%)	4 (14.8%)	1.000
Visual loss	7 (5.4)	5 (4.9%)	2 (7.4%)	0.633
Back low pain	6 (4.6)	4 (3.9%)	2 (7.4%)	0.603
Crackles	12 (9.2)	8 (7.8%)	4 (14.8%)	0.274

The data are the number of cases and the percentage.

**Table 2:** Quantitative characteristics of included patients.

Characteristics	Total (N = 130)	Without post COVID-19 sequels (N = 103)	Post COVID-19 sequels (N = 27)	MD (SE) CI 95%	t	*P*
Age, years	55.9 (15.8) (18.0–88.0)	53.4 (16.2) (18.0–88.0)	65.3 (9.9) (51.0–85.0)	11.869 (3.264) 5.408–18.329	3.635	< 0.001
Charlson index	1.9 (1.8) (0.0–9.0)	1.75 (1.8) (0.0–9.0)	2.7 (1.7) (0.0–7.0)	0.956 (0.388) 0.188–1.725	2.462	0.015
Length of hospitalization, days	6.5 (7.2) (0.0–34.0)	4.5 (5.7) (0.0–30.0)	14.0 (7.4) (0.0–34.0)	9.417 (1.326) 6.793–12.042	7.100	< 0.001
Pneumonia extensión at initial radiograph	3.9 (2.9) (0.0–10.0)	3.3 (2.8) (0.0–9.0)	6.2 (2.1) (3.0–10.0)	2.943 (0.586) 1.784–4.103	5.023	< 0.001
Pneumonia extensión at follow-up radiograph	1.1 (1.8) (0.0–9.0)	0.5 (1.2) (0.0–9.0)	3.3 (2.2) (0.0–7.0)	2.806 (0.316) 2.180–3.432	8.877	< 0.001
Pulmonary function						
FVC, mL	3847.0 (1073.4) (1550.0–7270.0)	3909.7 (1103.5) (1550.0–7270.0)	3613.1 (935.6) (1860.0–5250.0)		− 1.254	0.212
FVC, %	109.8 (17.1) (64.9–150.6)	109.8 (16.5) (64.9–145.5)	109.6 (19.7) (77.4–150.6)		− 0.036	0.972
FEV1, mL	2965.7 (931.1) (101.3–5530.0)	3020.3 (966.3) (101.3–5530.0)	2761.9 (768.5) (1310.0–4360.0)		− 1.260	0.210
FEV1, %	105.3 (20.8) (4.0–147.8)	104.7 (20.5) (4.0–147.7)	107.5 (22.2) (52.9–147.8)		0.617	0.538
FEV1/FVC %	77.2 (11.2) (54.0–122.0)	77.4 (11.9) (55.0–122.0)	76.5 (8.7) (54.0–88.0)		− 0.361	0.719

The data are the mean (standard deviation) and the range.

*MD* means differences, *SE* standard error, *CI 95%* 95% confidence interval for the difference in means, *FVC* forced vital capacity, *FEV1* forced expiratory volume in 1 second.

The predominant symptoms in both groups were dyspnea, asthenia and anosmia- ageusia. Spirometry was normal in both groups. We found significant differences between the two groups in age and in the Charlson index, with older age and more comorbidity in the group with pulmonary sequelae. In addition, we also found differences in hospital stay and in the extent of pneumonia, having stayed more days hospitalized and with a higher score in the group with sequelae.

Patients with pulmonary sequelae underwent additional studies with pulmonary diffusion and thoracic HRCT. Lung diffusion was normal with a mean DLCO of 79.50% (18.14) and range 55.0–108.0. Both spirometry and lung diffusion were redetermined at 12 months with the following results: mean FVC of 3690 ml (1013.0) and range 1430.0–5140.0, mean FVC% of 106.5% (14.3) and range 64.0–136.0, mean FEV1 of 2852.3 (766.9) and range 1190.0–4300.0, mean FEV1% of 105.6% (14.4%) and range 70.0–135.0, mean FEV1%FVC of 76.7% (7.8) and range 57.0–91.0 and mean DLCO% of 84.8 (14.4) and range 55.0–112.0. No significant differences were found in lung function tests at 3 and 12 months: FVC (p = 0.423), FVC% (p = 0.087), FEV1 (p = 0.233), FEV1% (p = 0.130), FEV1%FVC (p = 0.527), DLCO% (0.296).

The most frequently observed radiological findings in patients with alterations in CT were GGO, present in 88.9% of cases. However, the extension of the GGO was minimal or slight in most of the participants (55.5%). The peripheral distribution and in the middle and lower areas were the predominant locations. Reticulation (77.7%), fine parenchymal bands (63.0%), mosaic (40.7%) and distortion with traction bronchiectasis (29.6%) were the other alterations observed, but with minimal or slight extension. Severe radiological alterations were detected in 14.8% of the cases.

### Analysis of evolution and differences in HRQoL between patients with Post COVID-19 pulmonary sequelae and patients without post COVID-19 pulmonary sequelae

A general linear repeated measures model with the within-subject factor "Time" (Time 1: 3 months post infection and Time 2: 12 months post infection)" and the between-subject factor "Group" (patients with post infection sequelae and patients without post infection sequelae) was performed to analyse the evolution in the different dimensions of HRQoL, as well as possible differences according to group.

Regarding the variable Time, no differences were found between Time 1 (3 months post infection) and Time 2 (12 months post infection) in any of the HRQoL dimensions (p > 0.05), except in the case of the PCS dimension F(1,128) = 7.045, p = 0.009, n2partial = . 052 and MCS F(1,128) = 5.615, p = 0.019, n2partial = 0.042. In the case of PCS, at Time 2 participants obtained lower scores compared to Time 1; however, with respect to MCS, at Time 2 participants showed higher scores compared to Time 1.

Regarding the interaction "Time × Group", no significant interaction effect was found in most dimensions, except for GH F(1,128) = 8.761, p = 0.004, n2partial = 0.064. However, post-hoc analysis did not reveal significant differences in any specific time (p > 0.05).

Finally, no significant main effect of the factor "Group" was found (p > 0.05).

Table 3 shows the means and standard deviations of the scores obtained in each of the HRQoL dimensions both in the total sample and in each of the groups separately, taking into account the assessment time.

**Table 3:** Differences in the scores of the items of the SF-36 questionnaire at 3 and 12 months after infection.

HRQoL	Time 1 (3 months after the infection)			*p*	Time 2 (12 months after the infection)			MD (SE) CI 95%	*P*
	Total sample	Without post covid-19 sequels, n = 103	Post covid-19 sequels, n = 27		Total sample	Without post covid-19 sequels, n = 103	Post covid-19 sequels, n = 27		
Physical functioning	75.1 (23.1) (10.0–100.0)	75.5 (23.7) (10.0–100.0)	73.5 (20.9) (35.0–100.0)	0.695	70.7 (26.6) (0.0–100.0)	71.0 (27.3) (0.0–100.0)	69.9 (23.9) (20.0–100.0)	− 1.115 (5.763) (− 12.519–10.289)	0.847
Physical role	38.8 (44.5) (0.0–100.0)	37.9 (44.7) (0.0–100.0)	42.6 (44.3) (0.0–100.0)	0.625	41.2 (42.9) (0.0–100.0)	41.5 (42.8) (0.0–100.0)	39.8 (44.0) (0.0–100.0)	− 1.690 (9.308) (− 20.108–16.728)	0.856
Bodily pain	68.0 (27.0) (0.0–100.0)	68.3 (27.4) (0.0–100.0)	67.9 (27.5) (12.0–100.0)	0.955	60.6 (31.1) (0.0–100.0)	59.8 (30.9) (0.0–100.0)	63.7 (32.5) (12.0–100.0)	3.917 (6.751) (− 9.441–17.275)	0.563
General health	63.3 (21.6) (15.0–100.0)	63.9 (21.5) (15.0–100.0)	61.1 (23.1) (15.0–100.0)	0.561	57.2 (24.3) (5.0–100.0)	55.2 (24.2) (5.0–100.0)	64.9 (23.6) (25.0–97.0)	9.702 (5.206) (− 0.598–20.004)	0.065
Vitality	56.1 (27.2) (0.0–100.0)	54.6 (27.3) (0.0–100.0)	61.8 (27.2) (6.7–100.0)	0.233	54.7 (27.5) (0.0–100.0)	52.1 (27.4) (0.0–100.0)	64.3 (26.6) (5.0–100.0)	12.185 (5.883) 0.544–23.825	0.040
Social functioning	69.7 (29.9) (0.0–100.0)	69.2 (31.0) (0.0–100.0)	71.7 (25.6) (25.0–100.0)	0.690	74.5 (29.0) (0.0–100.0)	73.4 (29.6) (0.0–100.0)	78.7 (26.8) (12.5–100.0)	5.281 (6.279) (− 7.143–17.706)	0.402
Emotional role	50.7 (45.6) (0.0–100.0)	51.0 (46.5) (0.0–100.0)	49.3 (47.2) (0.0–100.0)	0.873	56.7 (46.7) (0.0–100.0)	54.7 (46.9) (0.00–100.0)	64.2 (46.2) (0.0–100.0)	9.505 (10.103) (− 10.468–29.496)	0.349
Mental health	68.6 (24.0) (0.0–100.0)	68.0 (24.9) (0.0–100.0)	71.2 (20.9) (16.0–100.0)	0.539	70.1 (23.8) (4.0–100.0)	67.9 (23.8) (4.0–100.0)	78.6 (22.0) (20.0–100.0)	10.709 (5.075) 0.666–20.752	0.037
Physical component summary score	44.9 (9.6) (14.9–73.1)	45.0 (9.8) (14.9–73.1)	44.4 (10.6) (24.9–69.6)	0.806	42.2 (11.6) (13.4–60.6)	42.3 (11.6) (13.4–60.6)	41.9 (11.4) (15.6–57.3)	− 0.375 (2.508) (− 5.338–4.588)	0.881
Mental component summary score	42.6 (14.7) (6.3–69.6)	42.3 (15.2) (6.3–69.6)	43.8 (14.7) (15.0–67.0)	0.667	45.0 (14.8) (12.1–68.0)	43.7 (14.7) (12.1–68.0)	49.8 (14.7) (22.1–66.0)	6.081 (3.175) (− 0.202–12.365)	0.058

The data are the mean (standard deviation) and the range. *MD* means differences, *SE*: standard error, *CI 95%* 95% confidence interval for the difference in means.

### Analysis of evolution and differences in HRQoL between patients with post COVID-19 pulmonary sequelae and patients without post COVID-19 pulmonary sequelae controlling for possible confounders

Considering that several differences have been identified in the characteristics of the sample at baseline, mainly regarding age, Charlson index, days of hospitalization and Pneumonia extension at initial radiograph; analyses of repeated measures were replicated but including these variables as covariates in the model.

In relation to the interaction "Time × Group", the significant effect of the interaction was maintained in the dimension GH F(1,124) = 6.313, p = 0.013, n2partial = 0.048. No significant effect of this interaction was found in any other dimension of HRQoL (p > 0.05).

Regarding the effect of the main factor "Group", differences were found in the dimension VT F(1,124) = 4.223, p = 0.042, n2partial = 0.033. For the remaining HRQoL dimensions, no significant main effect of the Group factor was found.

In relation to the covariates, days of hospitalization emerged significant in the between-subject effects in the following dimensions: FP F(1,124) = 4.453, p = 0.037, n2partial = 0.035; RP F(1,124) = 7.070, p = 0.009, n2partial = 0.054; PA F(1,124) = 6.435, p = 0.012, n2partial = 0.049; VT F(1,124) = 4.330, p = 0.039, n2partial = 0.034; SF F(1,124) = 6.137, p = 0.015, n2partial = 0.047; RE F(1,124) = 4.482, p = 0.036, n2partial = 0.035; MH F(1,124) = 4.548, p = 0.035, n2partial = 0.035; PCS F(1,124) = 4.022, p = 0.047, n2partial = 0.031 and MCS F(1,124) = 4.518, p = 0.036, n2partial = 0.035.

Charslon’s index was significant for between-subject effects in GH F(1,124) = 5.160, p = 0.025, n2partial = 0.040 and SF F(1,124) = 4.411, p = 0.038, n2partial = 0.034. In addition, it was significant for within-subject effects in the case of MH F(1,124) = 4.850, p = 0.029, n2partial = 0.038.

Radiological score at baseline was significant in between-subject effects in TV F(1,124) = 4.478, p = 0.036, n2partial = 0.035 and in MH F(1,124) = 4.662, p = 0.033, n2partial = 0.036.

### Institutional review board statement

The study was conducted in accordance with the Declaration of Helsinki and approved by the Ethics Committee of Investigación de Medicamentos del departamento de salud de Alicante (Dictamen Favorable PI2021-090 (ISABIAL 2021-0145)).

### Informed consent

Consent was obtained from all subjects involved in the study.

## Discussion

The emergence of COVID-19 disease in central China in December 2019, followed by the global outbreak in 2020 and its persistence to date, has become a global health, social and economic problem. Recovery time from the disease is variable but a significant percentage of patients have persistent symptoms, pulmonary sequelae and impaired quality of life.

While healing from COVID-19 pneumonia, CT-scans show minimal residual pulmonary abnormalities 1 year after the acute episode, with near complete resolution in 93% of the cases^10^. Ground glass opacities (GGO) distributed in the lower lobes is the main alteration of the disease and its prevalence decreases progressively, being 100% at the onset of the disease, 20% at 6 months, and 2% at 1 year. Fibrotic changes such as reticulation, traction bronchiectasis and/or bronchioloectasis are detectable in few patients (2%) during the initial evaluation and remain after the acute phase is resolved. Differently, other studies indicate a prevalence of residual lesions in 24–30% of the cases^9,15,30^ and associate it with the extent of the initial CT scan.

In line with the literature, the most frequently observed radiological alteration in our study was ground glass opacities (GGO) (89%), with peripheral distribution, and in the middle and lower zones of both hemithoraxes. In more than half of the cases (55%), these alterations were minimal or mild. In addition, the presence of residual changes was associated with a greater extent of initial disease and hospital stay. Both factors are described in a meta-analysis of variables related to the development of fibrosis after SARS-CoV-2 pneumonia^31^^.^

Patients with pulmonary sequelae after COVID-19 have been reported to have impaired lung function, with decreased FVC and DLCO^15,32^. This is confirmed by studies conducted at 3 months after hospital discharge which detect restrictive ventilatory impairment (decreased TLC) or decreased pulmonary diffusion in up to 52.5% of cases. Although at 6 months the parameters improved, only a minority normalised the tests and when thoracic HRCT was performed at 6 months, fibrotic areas associated with diffuse interstitial involvement were confirmed^33^.

In our study, patients with fibrotic radiological alterations did not show a decrease in forced vital capacity or pulmonary diffusion. This finding may be explained by the extent of the sequelae, which are minimal or mild. In other fibrosing lung diseases such as idiopathic pulmonary fibrosis, a disease in which pulmonary distensibility is substantially modified and the exchange area is reduced, the variables that best quantify these alterations would be the determination of forced vital capacity, total lung capacity (TLC) and DLCO. In the initial stages of the disease in which there is not much destructuring of the lung parenchyma, the functional tests are normal and as the disease progresses there is a restrictive ventilatory alteration with a decrease in FVC and TLC and also DLCO^34,35^.

COVID-19 pneumonia causes a deterioration in HRQoL. Several studies show that patients who survive SARS-CoV-2 pneumonia have poorer quality of life than the general population, with lower scores on all domains of the SF-36 questionnaire^2^. Moreover, this impairment persists over time, with deterioration 8 and 12 months after hospital discharge^2,36^.

Patients from the 2003 severe acute respiratory syndrome (SARS) epidemic exhibited different degrees of pulmonary fibrosis^17,18^ and an overall decline in most domains of the SF-36 questionnaire, these led to a worsening in the quality of life^19^. In contrast, very few data are available on the pulmonary sequelae of COVID-19 and their impact on HRQoL.

In our study, at 3 months, we found no differences between patients with and without pulmonary sequelae in the different domains of the questionnaire or in the physical and mental summary indices. At 12 months, patients without sequelae had lower scores in vitality and mental health. A general linear repeated measures model was performed to investigate the possible interaction between age, Charlson index, length of hospitalization and extent of pneumonia on the initial radiograph, factors in which differences were initially found between the two groups. Days of hospitalization was influential in almost all domains of the questionnaire and the Charlson index in general health and mental health. This finding confirms the negative impact that hospitalizations have on HRQoL, already demonstrated in other respiratory diseases^37^ and as in other systems^38^. In physical component summary score, physical function and physical role, a trend towards worse scores was detected in patients with pulmonary sequelae, although without reaching statistical significance.

The care received by patients with pulmonary sequelae may have influenced HRQoL, both because they have undergone more check-ups and medical examinations and because of the pulmonary rehabilitation they have received.

Many people who have overcome COVID-19 infection have persistent symptoms such as fatigue, dyspnea, gastrointestinal disturbances, mental confusion, and anxiety. Initially, these symptoms were neglected causing further suffering^39^ and patients who were initially less ill, virtually asymptomatic or managed by primary care during acute infection were most affected^40^. So much so that the World Health Organisation (WHO) recognized the long-lasting effects caused by the infection, forcing governments to recognize it and provide access to health services. The patients in our series have been monitored every 3 months and have received care and treatment for all symptoms, which has improved their health perception and comfort.

Patients with pulmonary sequelae in our series also received pulmonary rehabilitation. Pulmonary rehabilitation is a comprehensive intervention based on a thorough assessment of the patient, followed by tailor-made therapies including muscle training, education, and changes in lifestyle habits^41^. Treatment aims to improve the physical and psychological state of people with chronic respiratory diseases and has been the fundamental treatment strategy in patients with sequelae of COVID-19^22,23^. The correlation between quality of life and pulmonary rehabilitation in patients with COVID has already been demonstrated, even in mild to moderate infection^42^. Furthermore, a recent meta-analysis demonstrates a positive association between rehabilitation and lung function, exercise capacity and quality of life in patients with interstitial lung disease, including those severely affected by COVID-19^43^.

Taken together, these circumstances might have influenced the outcome of this study and explains why the participants of this study have better general health and mental health outcomes.

In terms of evolution, differences were only found in general health with opposite trends in the two groups. The group with pulmonary sequelae showed a trend towards improvement in all domains of the questionnaire except physical function, physical role, bodily pain and physical component summary score, although without reaching statistical significance. Other studies analyzing HRQoL evolution in the long term (3, 6 and 12 months) only found improvement in the physical role^44^. Published articles on the follow-up of patients with COVID-19 have been based on shorter follow-up periods, so there is very little data available to compare these results.

This study has some limitations. At 12 months, only 43% of all participants completed the questionnaire. This fact has reduced the sample size and may have contributed to the lack of significance in some of the long-term comparisons. Furthermore, we must consider the selective abandonment of participants, mainly due to lack of motivation, which has caused some patients who continue to be followed up to be those with the worst disease progression and require treatment. The low prevalence of radiological alterations (20%) should also be considered, as it might have influenced the lack of significance in the comparisons. In addition, another limitation of the study was the impossibility to compare CT scans. During follow-up, CT scans were performed at 6 and 12 months after hospital discharge. Initially, the radiology service reports on pulmonary changes were thorough and detailed. Due to the work overload and the lack of human and care resources, the CT scans performed at 12 months reported the alterations but in a coarser manner, which prevented a comparison between the two and an assessment of the evolution of the sequelae. In our study, the rehabilitation programme was adapted and personalised according to the specific needs of each patient, so the number of sessions and exercises were different from patient to patient. Although we do not consider this to be a real limitation, as different studies show the benefits of personalising health care and adapting treatments to individual needs, methodologically they have not received the same treatment^45–47^.

## Conclusions

In this study, we conclude that minimal or mild pulmonary sequelae of SARS-CoV-2 do not lead to further deterioration in HRQoL. Repeat medical care and pulmonary rehabilitation, considered as fundamental parts by the main guidelines on the management and treatment of patients with post-COVID-19 sequelae, seem to be also effective tools to improve HRQoL. Although the effectiveness of rehabilitation programmes has not been analysed in this study, the data show that it may be relevant to minimise the overall effect of the pathology and, consequently, improve patients’ general condition as well as their quality of life and lifestyle.

## Data Availability

The datasets generated during and/or analysed during the current study are not publicly available due to the preservation of the confidentiality and anonymity of participants, but are available from the corresponding author on reasonable request.

## References

[1] WHO. Coronavirus Disease (COVID-19) Dashboard. https://covid19.who.int/ (Accessed 21 January 2024).

[2] Rodríguez-Galán I, Albaladejo-Blázquez N, Ruiz-Robledillo N, Pascual-Lledó JF, Ferrer-Cascales R, Gil-Carbonell J (2022). Impact of COVID-19 on health-related quality of life: A longitudinal study in a spanish clinical sample. Int. J. Environ. Res. Public Health.

[3] Poudel AN, Zhu S, Cooper N, Roderick P, Alwan N (2021). Impact of Covid-19 on health-related quality of life of patients: A structured review. PLoS One.

[4] Malik P, Patel K, Pinto C, Jaiswal R, Tirupathi R, Pillai S, Patel U (2021). Post-acute COVID-19 syndrome (PCS) and health-related quality of life (HRQoL)-A systematic review and meta-analysis. J. Med. Virol..

[5] Boutou AK, Asimakos A, Kortianou E, Vogiatzis I, Tzouvelekis A (2021). Long COVID-19 pulmonary sequelae and management considerations. J. Pers. Med..

[6] Wu Z, McGoogan JM (2020). Characteristics of and important lessons from the coronavirus disease 2019 (COVID-19). Outbreak in China: Summary of a report of 72314 cases from the Chinese center for disease control and prevention. JAMA.

[7] Rubin GD, Ryerson CJ, Haramati LB (2020). The role of chest imaging in patient management during the COVID-19 pandemic: A multinational consensus statement from the Fleischner society. Chest.

[8] Nalbandian A, Sehgal K, Gupta A, Madhavan MV, McGroder C, Stevens JS, Cook JR, Nordvig AS, Shalev D, Sehrawat TS (2021). Post-acute COVID-19 syndrome. Nat. Med..

[9] Han X, Fan Y, Alwalid O (2021). Six-month follow-up chest CT findings after severe COVID-19 pneumonia. Radiology.

[10] Cortés-Telles A, López-Romero S, Figueroa-Hurtado E, Pou-Aguilar YN, Wong AW, Milne KM, Ryerson CJ, Guenette JA (2021). Pulmonary function and functional capacity in COVID-19 survivors with persistent dyspnoea. Respir. Physiol. Neurobiol..

[11] Shah AS, Wong AW, Hague CJ, Murphy DT, Johnston JC, Ryerson CJ, Carlsten C (2021). A prospective study of 12-week respiratory outcomes in COVID-19-related hospitalisations. Thorax.

[12] Wells AU, Devaraj A, Desai SR (2021). Interstitial lung disease after COVID-19 infection: A catalog of uncertainties. Radiology.

[13] Karampitsakos T, Akinosoglou K, Papaioannou O, Panou V, Koromilias A, Bakakos P, Loukides S, Bouros D, Gogos C, Tzouvelekis A (2020). Increased red cell distribution width is associated with disease severity in hospitalized adults with SARS-CoV-2 infection: An observational multicentric study. Front. Med..

[14] Bocchino M, Lieto R, Romano F, Sica G, Bocchini G, Muto E, Capitelli L, Sequino D, Valente T, Fiorentino G, Rea G (2022). Chest CT-based assessment of 1-year outcomes after moderate COVID-19 pneumonia. Radiology.

[15] Wu X, Liu X, Zhou Y (2021). 3-month,6-month,9-month, and12-month respiratory outcomes in patients following COVID-19-related hospitalisation: A prospective study. Lancet Respir. Med..

[16] Orme J (2003). Pulmonary function and health-related quality of life in survivors of acute respiratory distress syndrome. Am. J. Respir. Crit. Care Med..

[17] Hsu H-H (2004). Correlation of high-resolution CT, symptoms and pulmonary function in patients during recovery from severe acute respiratory syndrome. Chest.

[18] Herridge MS (2011). Functional disability 5 years after acute respiratory distress syndrome. N. Engl. J. Med..

[19] Hui DS, Wong KT, Ko FW, Tam LS, Chan DP, Woo J, Sung JJ (2005). The 1-year impact of severe acute respiratory syndrome on pulmonary function, exercise capacity, and quality of life in a cohort of survivors. Chest.

[20] der Sar-van Van, der Brugge S, Talman S, Boonman-de Winter L, de Mol M, Hoefman E, van Etten RW, De Backer IC (2021). Pulmonary function and health-related quality of life after COVID-19 pneumonia. Respir. Med..

[21] Valent A, Dudoignon E, Ressaire Q, Dépret F, Plaud B (2020). Three-month quality of life in survivors of ARDS due to COVID-19: A preliminary report from a French academic centre. Anaesth. Crit. Care Pain Med..

[22] NICE (2020). COVID-19 Rapid Guideline: Managing the Long-Term Effects of COVID-19.

[23] WHO. *Support for Rehabilitation: Self-Management after COVID-19-Related Illness*, 2nd edition, (WHO, 2021). https://apps.who.int/iris/bitstream/handle/10665/344472/WHO-EURO-2021-855-4059059892-eng.pdf?sequence=1&isAllowed=y (Accessed 7 August 2022).

[24] Sibila O, Molina-Molina M, Valenzuela C (2020). Documento de consenso de la Sociedad Española de Neumología y Cirugía Torácica (SEPAR) para el seguimiento clínico post-COVID-19. Open Respir. Arch..

[25] Litmanovich DE, Chung M, Kirkbride RR, Kicska G, Kanne JP (2020). Review of chest radiograph findings of COVID-19 pneumonia and suggested reporting language. J. Thorac. Imaging.

[26] Hansell DM, Bankier AA, MacMahon H, McLoud TC, Müller NL, Remy J (2008). Fleischner society: Glossary of terms for thoracic imaging. Radiology.

[27] Pellegrino R, Viegi G, Brusasco V (2005). Interpretative strategies for lung function tests. Eur. Respir. J..

[28] Alonso J, Prieto L, Anto JML (1995). versión española del SF-36 health survey (Cuestionario de Salud SF-36): Un instrumento para la medida de los resultados clínicos. Med. Clin..

[29] Vilaguta V, Ferrera M, El RL (2005). Cuestionario de Salud SF-36 español: Una década de experiencia y nuevos desarrollos. Gac. Sanit..

[30] Han X, Fan Y, Alwalid O (2021). Fibrotic interstitial lung abnormalities at 1-year follow-up CT after severe COVID-19. Radiology.

[31] Hama Amin BJ, Kakamad FH, Ahmed GS (2022). Post COVID-19 pulmonary fibrosis; a meta-analysis study. Ann. Med. Surg. (Lond.).

[32] Tarraso J, Safont B, Carbonell-Asins JA (2022). Lung function and radiological findings 1 year after COVID-19: A prospective follow-up. Respir. Res..

[33] Orzes N, Pini L, Levi G, Uccelli S, Cettolo F, Tantucci C (2021). A prospective evaluation of lung function at three and 6 months in patients with previous SARS-COV-2 pneumonia. Respir. Med..

[34] Kirtland SH, Winterbauer RH (1997). Pulmonary function tests and idiopathic pulmonary fibrosis. Simple may be better. Chest.

[35] Casan Clarà P, Martínez González C, Ancochea J (2016). Lung function testing in idiopathic pulmonary fibrosis: More than just spirometry?. Arch. Bronconeumol..

[36] Aranda J, Oriol I, Martín M, Feria L, Vázquez N, Rhyman N, Vall-Llosera E, Pallarés N, Coloma A, Pestaña M (2021). Long-term impact of COVID-19 associated acute respiratory distress syndrome. J. Infect..

[37] Esteban C, Quintana JM, Moraza J (2009). Impact of hospitalisations for exacerbations of COPD on health-related quality of life. Respir. Med..

[38] Jalal SM, Beth MRM, Bo Khamseen ZM (2022). Impact of hospitalization on the quality of life of patients with chronic kidney disease in Saudi Arabia. Int. J. Environ. Res. Public Health.

[39] Lancet T (2020). Facing up to long COVID. Lancet.

[40] Norton A, ISARIC and GloPID-R Long COVID Forum Working Group (2021). Long COVID: Tack- ling a multifaceted condition requires a multidisciplinary approach. Lancet Infect. Dis..

[41] Spruit MA, Singh SJ, Garvey C, Zu Wallack R, Nici L, Rochester C (2013). An official American thoracic society/European respiratory society statement: Key concepts and advances in pulmonary rehabilitation. Am. J. Respir. Crit. Care Med..

[42] Levi G, Scaramozzino MU, Cavallo S, Castignini G, Bezzi M, Pini L, Nania F, Sheenam S (2023). Pulmonary rehabilitation improves functional outcomes and quality of life in post-SARS-CoV-2 mild-to-moderate infection patients: A pilot study. Monaldi Arch. Chest Dis. Arch. Monaldi Malattie Torace.

[43] Reina-Gutiérrez S, Torres-Costoso A, Martínez-Vizcaíno V, Núñez de Arenas-Arroyo S, Fernández-Rodríguez R, Pozuelo-Carrascosa DP (2021). Effectiveness of pulmonary rehabilitation in interstitial lung disease, including coronavirus diseases: A systematic review and meta-analysis. Arch. Phys. Med. Rehabil..

[44] Eberst G, Claudé F, Laurent L, Meurisse A, Roux-Claudé P, Barnig C, Vernerey D, Paget-Bailly S, Bouiller K, Chirouze C (2022). Result of one-year, prospective follow-up of intensive care unit survivors after SARS-CoV-2 pneumonia. Ann. Intensive Care.

[45] Wouters EFM, Wouters BBREF, Augustin IML, Houben-Wilke S, Vanfleteren LEGW, Franssen FME (2018). Personalised pulmonary rehabilitation in COPD. Eur. Respir. Rev. Off. J. Eur. Respir. Soc..

[46] Huo H, Wang Q, Zhou S, Cui L (2021). The application of personalized rehabilitation exercises in the postoperative rehabilitation of breast cancer patients. Ann. Palliat. Med..

[47] Spaulding W, Deogun J (2011). A pathway to personalization of integrated treatment: Informatics and decision science in psychiatric rehabilitation. Schizophr. Bull..

